# Hydroxysafflor yellow A attenuates the expression of inflammatory cytokines in acute soft tissue injury

**DOI:** 10.1038/srep40584

**Published:** 2017-01-11

**Authors:** Fang Dong, Changjiang Xue, Yu Wang, Yuanyuan Peng, Yadan Zhang, Ming Jin, Baoxia Zang

**Affiliations:** 1 Department of Pharmacology, Beijing Anzhen Hospital, Capital Medical University, Beijing Institute of Heart Lung and Blood Vessel Disease, Beijing, P.R. China

## Abstract

We examined the effect of hydroxysafflor yellow A (HSYA) on the inflammatory response to strike-induced acute soft tissue injury in rats. Soft tissue injury was induced in rat leg muscles using a strike hammer, followed by intraperitoneal administration of HSYA at 16, 32, or 64 mg/kg. After 24 h, the rats were anaesthetized, blood and muscle samples were taken. Plasma levels of interleukin (IL)-6, IL-1β, and tumour necrosis factor (TNF)-αwere measured by enzyme-linked immunosorbent assay. Total RNA and protein were isolated from muscle tissue to determine the mRNA levels of *IL-6, IL-1β, TNF-α*, vascular cell adhesion molecule (*VCAM)-1*, and intercellular adhesion molecule (*ICAM)-1*, and the protein level of phosphorylated p38 mitogen-activated protein kinase (MAPK). Nuclear factor (NF)-κB expression was determined by muscle histopathology and immunohistochemistry. HSYA attenuated pathologic changes instrike-induced soft tissue inflammation. Treatment with HSYA also alleviated strike-induced increases in *TNF-α, IL-1β, IL-6, VCAM-1*, and *ICAM-1*mRNA levels and inhibited the increased activation of NF-κB and phosphorylation of p38 MAPK in muscle tissue. These findings suggest that HSYA effectively inhibits strike-induced inflammatory signal transduction in rats.

Acute soft tissue injury is a common clinical exercise injury with significant impacts on people’s health and ability to work, and as such has been the focus of increasing attention. Aseptic inflammation is the most important pathological change occurring after acute soft tissue damage^1^. The damage can be manifested in many ways, including local oedema, muscle fibre fracture, pain, bruising, petechiae, and dysfunction.

Dried safflower (*Carthamus tinctorius* L.) flowers is a traditional Chinese medicine that has been used to promote blood circulation for over 2000 years. It has a long history of treating motor-system damage in China. It is traditionally used as a decoction for internal application, or as an external application. Safflower comprises >200 compounds, including flavonoids, phenylethanoid glycosides, coumarins, fatty acids, steroids, and safflower polysaccharides, of which hydroxysafflor yellow A (HSYA) is the effective flavonoid^2^ (Fig. 1). HSYA alleviates platelet aggregation and inhibits thrombosis^3^. The inhibitory effect of HSYA on pulmonary inflammation in mice^4^ and its attenuating effect on lipopolysaccharide-induced inflammatory signal transduction in human alveolar epithelial A549 cells have recently been reported^5^. However, to the best of our knowledge, there have been no reports on the use of HYSA for the treatment of acute soft tissue injury. The present study was therefore conducted to determine if HSYA could inhibit the strike-induced inflammatory response in acute soft tissue injury in rats.

## Results

### Muscle morphology

We investigated changes in tissue morphology associated with strike injury and HSYA treatment in rats by examining the surfaces of the fur and muscle (Fig. 2) and by hematoxylin-eosin (HE) staining of muscle tissue sections. There was no bleeding, gore, or oedema in the sham or HSYA blank group (Fig. 2; see experimental design in Methods). Serious bleeding, gore, and oedema were seen in the strike group, but these injuries were attenuated in the strike+HSYA and strike+dexamethasone (DXM) groups. To elucidate the effect of HSYA on muscle histopathological changes and cellular infiltration associated with strike-induced soft tissue injury, sections of left-leg muscle were stained and observed. The sham group showed normal muscle fibre arrangement and muscle cell shape. However, severe soft tissue inflammatory response in the strike group was associated with obvious infiltration of inflammatory cells, muscle fibre breaks and swelling, widening of the intermuscular septum, and muscle fibre degeneration. The soft tissue inflammatory response was attenuated in dose-dependent manners in the HSYA and DXM groups (Fig. 2).

### Effects of HSYA on mRNA expression levels of interleukin-6 (IL-6), interleukin-1 (IL-1), tumour necrosis factor-α (TNF-α), vascular cell adhesion molecule-1 (VCAM-1), and intercellular adhesion molecule-1 (ICAM-1)

Real-time quantitative polymerase chain reaction (RT-qPCR) was performed to evaluate the expression of major inflammatory factors in muscle homogenates after strike-induced acute soft tissue injury and HSYA treatment. mRNA levels of *TNF-α, IL-1β, IL-6, VCAM-1*, and *ICAM-1*in muscle tissue were significantly elevated in the strike group compared with the sham group (Fig. 3). This augmented expression was attenuated by HSYA in a dose-dependent manner. A similar effect was observed in the DXM group (Fig. 3).

### Effects of HSYA on plasma IL-6, IL-1β, and TNF-α protein concentrations in rats

Plasma IL-6, IL-1β, and TNF-α protein levels were significantly increased in the strike group compared with the sham group, but were attenuated in the strike+ HSYA and strike+DXM groups (Fig. 4).

### Effects of HSYA on p38 mitogen-activated protein kinase (MAPK) activation

p38MAPK is a known intracellular signal transducer of the inflammatory reaction, which can be triggered by various extracellular stimuli. p38 MAPK activation in muscle tissue after striking and HSYA treatment were assessed by western blot analysis. The level ofphosphorylated p38 MAPK was markedly augmented int he strike group, and this augmentation was attenuated by HSYA and DXM (Fig. 5).

### Effect of HYSA onnuclear factor (NF)-κB p65

We assessed the expression level of NF-κB subunit p65 by immunohistochemistry to determine if the observed anti-inflammatory effect of HSYA involved inhibition of NF-κB activity. The proportion of p65-positive cells was significantly increased in the strike group compared with the sham group, and treatment with HSYA or DXM significantly attenuated the strike-induced increase in p65-positive cells (Fig. 6).

## Discussion

Acute soft tissue injury is a common orthopaedic condition^6^. It is characterised by a series of acute contusions and/or tears in tissues beneath the skin, including muscle, ligament, fascia, tendon, synovium, fat, joint capsule peripheral nerves, and blood vessels, but excluding bones. Acute soft tissue injuries are generally induced by external stress above a threshold, causing symptoms such as localized swelling, pain, dysfunction, and bruising. The main pathological change in acute soft tissue injury involves traumatic aseptic inflammation, characterized by local tissue necrosis, blood capillary dilation, leukocyte infiltration, oedema, and haemorrhage.

An acute soft tissue injury model induced by a blunt blow has recently been used widely to study the anti-inflammatory effects of numerous drugs^7^,^8^. Although its aetiology differs from that of clinical acute soft tissue injuries, its pathology and clinical pathological manifestations are similar^7^. In the current study, we used a self-made blunt-blow device to establish a rat model of acute soft tissue injury to simulate the occurrence of clinical acute soft tissue injury and to examine the therapeutic effect HSYA on such injuries. Corticosteroids are commonly used for treating acute and chronic soft tissue injuries in the clinic^9^, and we therefore used DXM as a positive control drug to evaluate the efficacy of HSYA for the treatment of acute soft tissue injury.

Local swelling and bruising are the main manifestations of acute soft tissue injury, and are the basis for vascular-lesion rupture or damage, oozing red blood cells, inflammatory cells, and muscle fibre breaks, degeneration, and necrosis. Using HE staining, we showed that the muscle fibre structure was destroyed and there was severe skeletal muscle tissue oedema following trauma in our rat model, while HSYA relieved the oedema and bleeding.

Blunt blow causes an aseptic inflammatory response, fibroblast proliferation, and subsequent collagen deposition. Neutrophils also play an important role in the pathogenesis of acute soft tissue injury and are released early from traumatic lesions to induce free radicals, which subsequently damage cells in local tissues. Neutrophils release numerous inflammatory mediators, including TNF-α, IL-1β, IL-6, IL-8, IL-12, vasoactive amines, and arachidonic acid metabolites, which further exacerbate the adhesion and aggregation of inflammatory cells and the release of inflammatory factors, and concurrently activate new inflammation. TNF-α and IL-1β are two of the major proinflammatory cytokines involved in the early inflammatory response, causing a variety of biochemical effects^10^,^11^. IL-6 promotes neutrophil activation and aggregation, and IL-6 levels partially reflect the intensity of tissue injury^12^. Increasesin TNF-α and IL-1β induce the expression of adhesion molecules such as ICAM-1, resulting inlymphocyte adhesion to the endothelial cell surface^13^. HSYA may relieve blood circulation disorders^14^, and Safflor Yellow (the main ingredient of which is HSYA) can inhibit the increased blood capillary permeability caused by inflammation damage^15^. Leukocyte activation^16^, white blood cell adhesion to epithelial cells^5^, and endothelial cell adhesion molecule expression^17^ can all be inhibited by HSYA, and leukocyte infiltration was demonstrated in the current experiment. The results of the current study showed that strike-induced acute soft tissue injury in rats enhanced the mRNA and protein expression levels of TNF-α, IL-1β, IL-6, ICAM-1, and VCAM-1, and these were all significantly inhibited by HSYA treatment. In addition, the HYSA-induced decrease in inflammatory cytokine expression was associated with reduced oedema and bleeding.

Inflammatory mediators (e.g., TNF-α and IL-1β) can activate multiple intracellular signalling molecules, such as MAPK and NF-κB, and thus trigger the expression of related genes^18^,^19^. During acute soft tissue injury, skeletal muscle endothelial cells, macrophages, and neutrophils secrete a variety of proinflammatory cytokines and proteases that take part in early inflammation through the NF-κB and MAPK pathways. In this study, the proportion of p65-positive cells in muscle was significantly increased by striking, and significantly attenuated by treatment with HSYA or DXM. We therefore hypothesized that HSYA inhibited the NF-κB signalling pathway in skeletal muscle tissue. HSYA treatment also significantly decreased strike-induced p38 MAPK phosphorylation in skeletal muscle, as demonstrated by western blotting, indicating that the effect of HSYA may be related to inhibition of the MAPK pathway.

In summary, HSYA reduced strike-triggered local oedema and neutrophil infiltration in skeletal muscle in a dose-dependent manner. The mechanism of HYSA action appears to involve inhibition of p38 MAPK phosphorylation and suppression of NF-κB pathway activation, thus decreasing the gene and protein expression levels of TNF-α, IL-1β, IL-6, ICAM-1, VCAM-1, and other inflammatory mediators.

## Methods

### Chemicals and reagents

**Figure 1:**
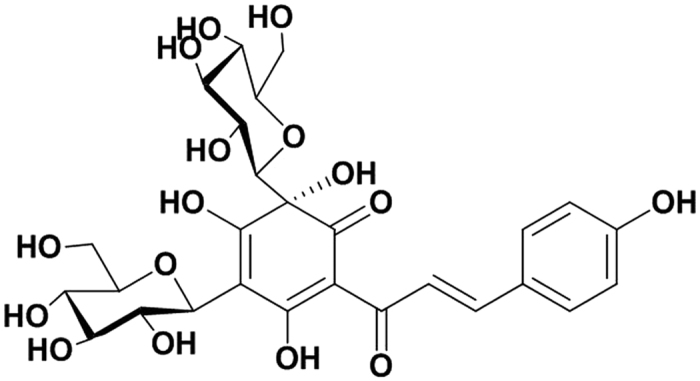
Molecular structure of hydroxysafflor yellow A.

**Figure 2:**
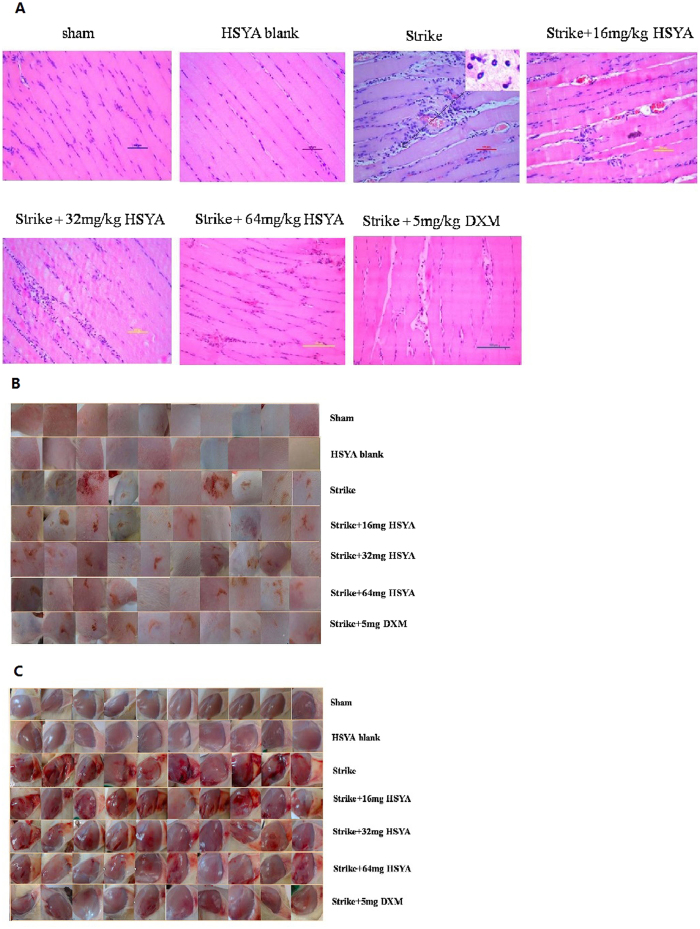
Effect of HSYA on morphological changes in stricken skeletal muscle. (**A**) HE staining 200×; in the strike group, tissue edema can be seen, and the inflammatory cell infiltration can be seen in the enlarged image. (**B**) Surface of rat fur after strike; (**C**) surface of muscle after strike.

**Figure 3:**
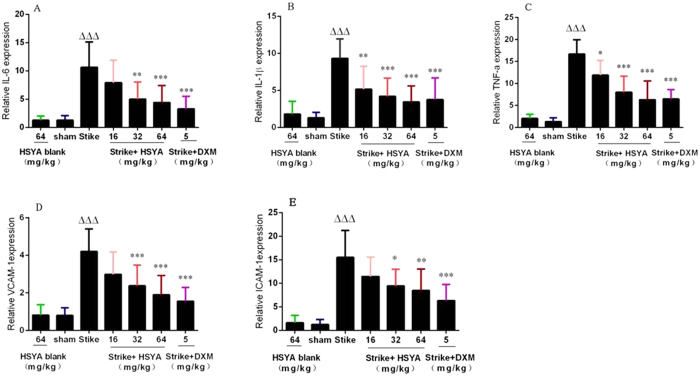
Effects of HSYA on *IL-6, IL-1β, TNF-α, VCAM-1*, and *ICAM-1* mRNA levels in rat muscle after acute soft tissue injury. Data are presented as mean ± standard deviation (SD); n = 10; *p < 0.05, *p < 0.01, ***p < 0.001 vs strike group. There was no significant difference between the sham control and HSYA blank groups.

**Figure 4:**
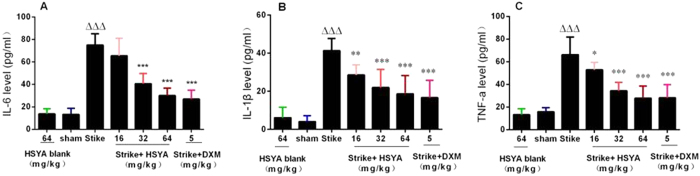
Effect of HSYA on IL-6, IL-1β, and TNF-α levels in rat plasma. Data are presented as mean ± SD; n = 10; ^*∆∆∆*^*p* < 0.001 vs. sham group, *p < 0.05, **p < 0.01, ***p < 0.001 vs strike group. There was no significant difference between the sham control and HSYA blank groups.

**Figure 5:**
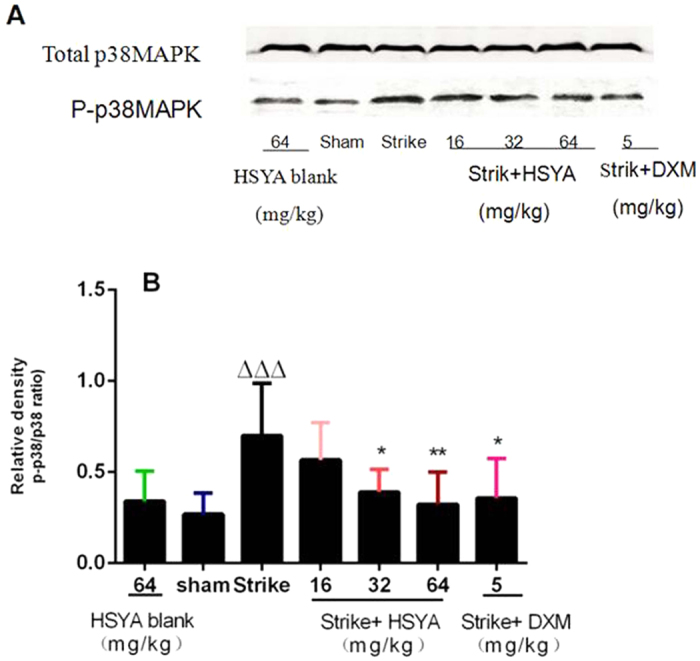
Western blot analysisofinhibitory effect of HSYA on muscle p38MAPK phosphorylation after acute soft tissue injury. ^ΔΔΔ^*p* < 0.001 vs. sham group, *p < 0.05, **p < 0.01 vs. strike group (n = 8).

**Figure 6:**
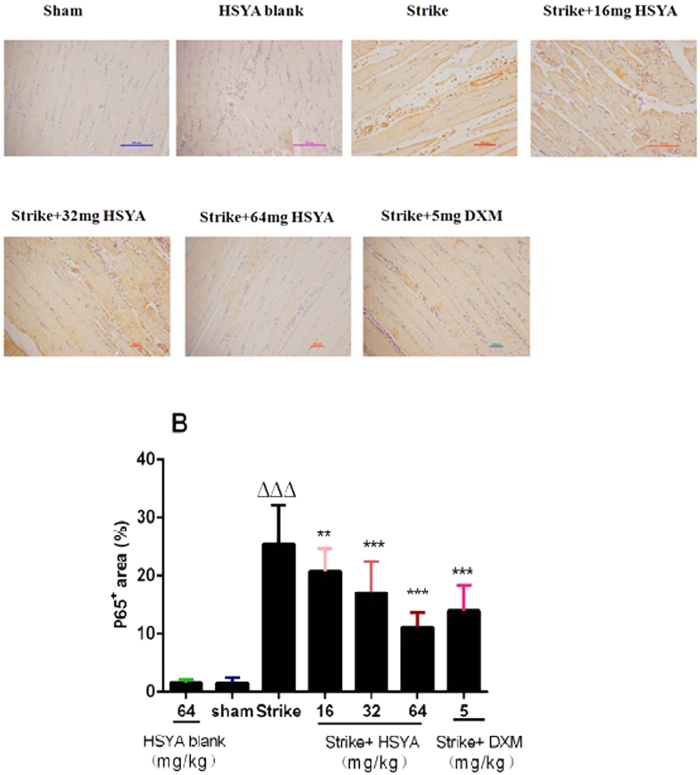
Effect of HSYA on NF-κBp65 level in rat skeletal muscle with acute soft tissue injury. (**A)** Muscle sections were examined by immunohistochemical staining (100×). (**A**) p65-positive cells are stained brown. (**B**) Quantification of NF-κB p65 staining. ^ΔΔΔ^*p* < 0.001 vs. sham group, *p < 0.05, **p < 0.01, ***p < 0.001 vs strike group (n = 8).

**Figure 7:**
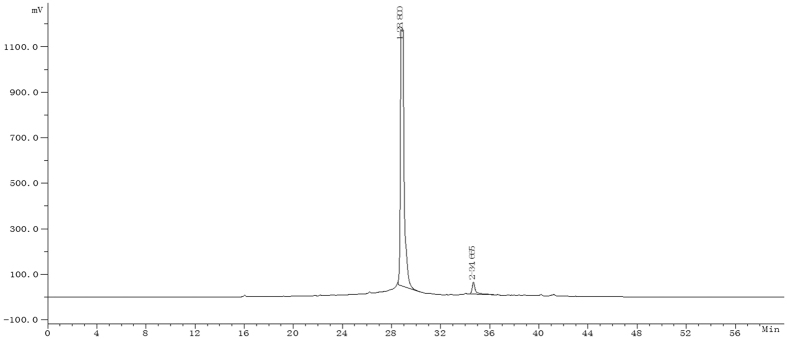
HPLC analysis of HSYA. The absorbance was measured at 405 nm. The peak at 28 min indicates HSYA. The purity of HSYA was 95.9%.

Dried safflower (*C. tinctorius* L.) flowers were obtained from Tacheng, Xinjiang Uygur Autonomous Region, China, and identified by Professor Jiashi Li (Beijing University of Chinese Medicine). HSYA was isolated and purified from aqueous extracts of *C. tinctorius* L. by macroporous resin-gel column chromatography, as described previously^20^, with a purity of 95.9% determined by high-performance liquid chromatography (HPLC) (Fig. 7). HSYA was dissolved insterile 0.9% NaCl for subsequent use. The molecular weight of HSYA was 612, and its molecular structure is displayed in Fig. 1. TRIzol reagent was purchased from Life Technologies (Carlsbad, CA, USA), RNasin Ribonuclease Inhibitor, Oligo dT, and M-MLV Reverse Transcriptase were from Promega (Madison, WI, USA), SYBR^®^ Premix Ex Taq™ (Perfect Real Time) kit was from Takara Bio Inc. (Otsu, Shiga, Japan), and enzyme-linked immunosorbentassay (ELISA) kits were purchased from ShanghaiExCellBiology, Inc. (Shanghai, China). Antibodies against p38 MAPK, phospho-p38MAPK, and NF-κB p65, were purchased from Cell SignalingTechnology (Danvers, MA, USA).

### HPLC analysis of HSYA

HPLC analyses were performed with an Apollo C18 column (250 mm × 4.6 mm, 5 μm; Grace Davison (Curtis Bay, MD, USA)) on an LC-10AT HPLC system with an SPD-6AV UV detector (Shimadzu, Kyoto, Japan). The mobile phase consisted of acetonitrile (A) and 0.1% trifluoroacetic acid (B) at a flow rate of 1.0 mL/min. The gradient elution program was as follows: initial 1% solvent A and 99% solvent B; from 0–50 min, solvent A was linearly increased from 1% to 35%, and solvent B was linearly decreased from 99% to 65%; from 50–60 min, solvent A was linearly increased from 35% to 45%, and solvent B was linearly decreased from 65% to 55%. The optical absorbance was monitored at 405 nm and the column temperature was 30 °C. The HSYA purity was determined quantitatively by the area normalization method.

### Animals

Pathogen-free mature male Wistar rats weighing 200–240 g were obtained from the Centre of Experiment Animals of Vital River Laboratories (Beijing, China). Animals were maintained in the Animal Department of Anzhen Hospital under controlled temperature (23 ± 2 °C) and humidity (60 ± 10%) conditions, with a 12 h light/dark cycle. All animal experiments were performed in compliance with the guidelines of Beijing Laboratory Animal Management Ordinance and approved by the Institutional Animal Care and Use Committee of Capital Medical University.

### Experimental design and treatment

**Figure 8:**
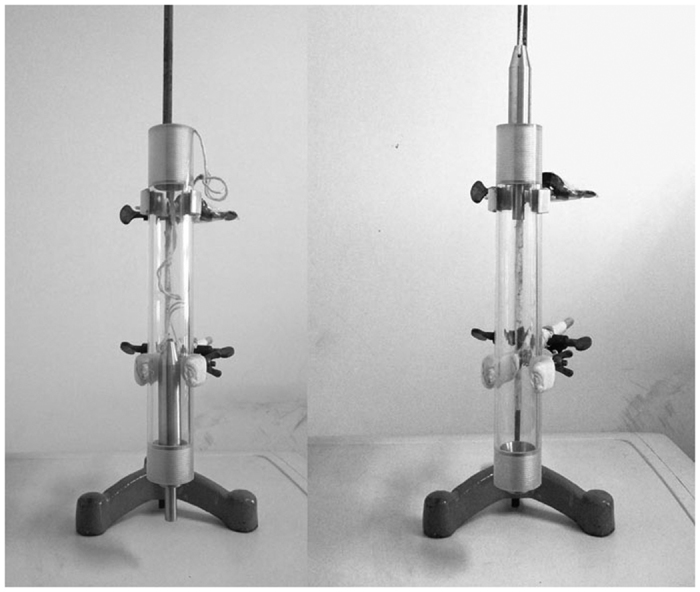
Self-made hammer device.

Eighty-four Wistar rats were divided randomly into seven groups: sham group (saline 64 mg/kg, intraperitoneal [i.p.]), HSYA blank group (HSYA 64 mg/kg, i.p.), strike group (strike + saline), strike+HSYA groups (strike + HSYA 16, 32, or 64 mg/kg, i.p.), and strike + DXM group (strike +DXM 5 mg/kg, i.p.). All rats were anaesthetized with 1% pentobarbital i.p. The medial thigh 1 cm from the knee joint was depilated with depilatory cream. A soft tissue injury model was established using a self-made hammer as follows: a stainless steel hammer weighing 267 g, with a bottom-surface radius of 0.5 cm (Fig. 8), was dropped through the central vertical axis of a plastic tube from a height of 30 cm to hit the medial thigh, 1 cm from the knee joint. In the sham groups, a marker pen was used instead of the stainless steel hammer. Rats in the sham and strike groups were injected i.p. twice with sterile 0.9% NaCl at 30 min and 6 h after the strike. Rats in the strike+HSYA and strike+ DXM groups were then injected i.p. with the indicated doses of HSYA or DXM, respectively. At 24 h after the strike, the rats were anaesthetized with pentobarbital sodium, blood samples were obtained from the abdominal aorta using a heparinised syringe for plasma preparation, and the rats were sacrificed by exsanguination. Injured muscle tissues were cut and washed in ice-cold saline, and muscle tissues from the right leg were snap-frozen in liquid nitrogen for RNA isolation and protein extraction. Muscle tissues from the left leg were trimmed and fixed in 4% paraformaldehyde solution for histopathological and immunohistochemical examinations.

### Histopathology studies

Muscle tissues from the left leg were fixed in 4% paraformaldehyde solution, and embedded in paraffin. Sequential 5-μm sections were stained with HE. Slides were scanned and images were taken under a light microscope (Nikon Eclipse 90i, Tokyo, Japan).

### Immunohistochemical detection

After treatment with xylene and hydration with graded alcohols, the muscle samples were incubated in citrate buffer (pH 6.0) at 96 °C for 20 min for antigen retrieval. Samples were washed three times in phosphate-buffered saline (PBS), blocked with rabbit serum for 30 min, and then incubated with primary antibody against p65 (1:100) at 4 °C overnight. After washing with PBS, the samples were incubated in biotinylated rabbit anti-goat antibody for 60 min at 37 °C. Some sections were also incubated exclusively with primary antibody or exclusively with secondary antibody to verify the binding specificity, which confirmed no positive staining in these sections. Digital images were captured at 100× magnification from five randomly selected fields for each section, and positive areas were integrated using the NIS-ELEMENTS quantitative automatic program (Nikon). The average optical absorbance was regarded as the level of target protein.

### RT-PCR analysis

Total RNA was isolated from muscle tissue using TRIzol reagent, according to the manufacturer’s instructions. RNA quality and concentration were assayed using a NanoDrop 2000 device (Thermo Scientific, Wilmington, DE, USA). The mRNA was then reverse transcribed into cDNA using an RT-PCR kit. Target mRNA was quantified by real-time PCR using the SYBR^®^ Premix Ex Taq™ kit on a Bio-Rad iCycler iQ5 Real-time Detection System (Hercules, CA, USA). The PCR amplification conditions were as follows: initial denaturation at 95 °C for 30 s, followed by 40 cycles of denaturation at 95 °C for 15 s and annealing at 60 °C for 30 s. The sequences of the respective sense and antisense primers were as follows: *β-actin,* 5′-TGGCATCCACGAAACTACCT-3′ and 5′-TCAGGAGCAATGATCTTG -3′; *IL-6*, 5′-GCTCTGGTCTTCTGGAGTTCC-3′ and 5′-GAGTTGGATGGTCTTGGTCCT-3′; *IL-1β*, 5′-ACAAGGAGAGACAAGCAACGA-3′ and 5′-TCTGCTTGAGAGGTGCTGATG-3′;*TNF-α*, 5′-GCCAATGGCATGGATCTCAA-3′ and 5′-ACTTGGGCAGGTTGACCTCA-3′; *ICAM-1*, 5′-TCCGGTAGACACAAGCAAGAG-3′ and 5′-AGAAGCCCAAACCCGTATGA-3′; and *VCAM-1*, 5′-GGATGCCGGAGTATACGAGTG-3′ and 5′-CTTCTGTGCCTCCACCAGACT-3′. Relative mRNA levels were calculated using the 2^−ΔΔCt^ method. All the results were normalised to the level of *β-actin*mRNA.

### Western blot analysis

Phosphorylated p38 MAPK levels in muscle tissues were determined by western blot analysis. Protein concentration was determined using the bicinchoninicacid method, and equal amounts were loaded on a 12% sodium dodecyl sulphate-polyacrylamide gel for electrophoresis. Protein bands were then transferred onto nitrocellulose membranes and blocked with Tris-buffered saline 0.1% Tween 20 (TBST) containing 5% non-fat dried milk. The membranes were incubated with primary antibodies overnight at 4 °C. Mouse monoclonal anti-p38 MAPK antibody and anti-phospho-p38 MAPK antibody were diluted 1:1000. Following incubation, the membranes were washed three times with TBST, followed by incubation with horseradish peroxidase-conjugated goat anti-rabbit antibody (diluted 1:1000 in TBS). Reactive proteins were detected using a chemiluminescent solution, and the bands were quantified using an Odyssey Infrared Imaging System (Lincoln, NE. US). The internal control for phospho-p38 MAPK was p38 MAPK.

### ELISA

Blood samples were obtained from the abdominal aorta using a heparinised syringe, and centrifuged at 1100 × *g* for 10 min to prepare plasma. TNF-α, IL-1β, and IL-6 concentrations in the plasma were measured by ELISA, according to the manufacturer’s protocols.

### Statistical analysis

All data are expressed as mean ± SD. Statistical analyses were performed using one-way analysis of variance with two-tailed tests and Student–Newman–Keuls multiple comparison tests, using SPSS 19.0 software. Figures were generated using GraphPad Prism 5.0 software. A value of *p* < 0.05 indicated a significant difference.

## Additional Information

**How to cite this article**: Dong, F. *et al*. Hydroxysafflor yellow A attenuates the expression of inflammatory cytokines in acute soft tissue injury. *Sci. Rep.* **7**, 40584; doi: 10.1038/srep40584 (2017).

**Publisher's note:** Springer Nature remains neutral with regard to jurisdictional claims in published maps and institutional affiliations.
